# *‘We’re passengers sailing in the same ship*, *but we have our own berths to sleep in’*: Evaluating patient and public involvement within a regional research programme: An action research project informed by Normalisation Process Theory

**DOI:** 10.1371/journal.pone.0215953

**Published:** 2019-05-14

**Authors:** Julia Keenan, Fiona Poland, Jonathan Boote, Amanda Howe, Helena Wythe, Anna Varley, Penny Vicary, Lisa Irvine, Amander Wellings

**Affiliations:** 1 School of Health Sciences, University of East Anglia, Norwich Research Park, Norwich, United Kingdom; 2 School of Health and Related Research, University of Sheffield, Sheffield, United Kingdom; 3 Centre for Research in Primary and Community Care, University of Hertfordshire, Hatfield, Hertfordshire, United Kingdom; 4 School of Medicine, University of East Anglia, Norwich Research Park, Norwich, United Kingdom; 5 Public and Patient Involvement in Research (PPIRes), South Norfolk Clinical Commissioning Group, Broadland Business Park, Norwich, United Kingdom; Foundation IRCCS Neurological Institute C. Besta, ITALY

## Abstract

**Background:**

Patient and public involvement (PPI) is a requirement for UK health and social care research funding. Evidence for how best to implement PPI in research programmes, such as National Institute for Health Research (NIHR) Collaborations for Applied Health Research and Care (CLAHRCs), remains limited. This paper reports findings from an action research (AR) project called IMPRESS, which aims to strengthen PPI within CLAHRC East of England (EoE). IMPRESS combines AR with Normalisation Process Theory (NPT) to explore PPI within diverse case study projects, identifying actions to implement, test and refine to further embed PPI.

**Methods:**

We purposively selected CLAHRC EoE case study projects for in-depth analysis of PPI using NPT. Data were generated from project PPI documentation, semi-structured qualitative interviews with researchers and PPI contributors and focus groups. Transcripts and documents were subjected to abductive thematic analysis and triangulation within case. Systematic across case comparison of themes was undertaken with findings and implications refined through stakeholder consultation.

**Results:**

We interviewed 24 researchers and 13 PPI contributors and analysed 28 documents from 10 case studies. Three focus groups were held: two with researchers (n = 4 and n = 6) and one with PPI contributors (n = 5). Findings detail to what extent projects *made sense* of PPI, *bought in to* PPI, o*perationalised* PPI and *appraised* it, thus identifying barriers and enablers to fully embedded PPI.

**Conclusion:**

Combining NPT with AR allows us to assess the embeddedness of PPI within projects and programme, to inform specific local action and report broader conceptual lessons for PPI knowledge and practice informing the development of an action framework for embedding PPI in research programmes. To embed PPI within similar programmes teams, professionals, disciplines and institutions should be recognised as variably networked into existing PPI support. Further focus and research is needed on sharing PPI learning and supporting innovation in PPI.

## Introduction

Patient and Public Involvement (PPI) is health research policy in the United Kingdom (UK) and internationally [1–4]. PPI in research is defined as research undertaken *with*, or *by*, patients and the public, rather than research which is *on*, *for*, or *about* them [5]. A contrast is drawn between involvement and participation in research; those involved are not participants in the research but rather advisors, co-researchers or co-applicants. Researchers and PPI contributors can work together during various phases of the research cycle: to prioritise, plan, conduct and disseminate research. PPI is also practiced in setting research priorities and in funding allocation.

In England, The National Institute for Health Research (NIHR) supports PPI across a wide range of organisations, including Research Design Services, Local Clinical Research Networks and Biomedical Research Centres and Units and, of particular interest here, Collaborations for Leadership in Applied Health Research and Care (CLAHRCs). As with similar initiatives worldwide [6] CLAHRCs in England’s regions seek to address the ‘second translational gap’ between identifying and evaluating new effective interventions appropriate for everyday use, and implementing them in NHS practice [7]. CLAHRCs were established to accelerate translation, through partnerships between health-care organisations and universities focused on improving patient outcomes by conducting and applying research [8]. CLAHRCs typically have several research ‘themes’ reflecting and serving local (regional) needs by being community-focused, and actively involving patients, public and other relevant stakeholders [8]. Within the broader NIHR infrastructure CLAHRCs are expected to play a key leadership role across PPI networks [4] by identifying cross-cutting activity in public involvement, developing joint plans and stable resourcing as members of wider emergent and evolving regional PPI networks. While numerous benefits of PPI in research have been reported (see below), so too have challenges for ‘scaling up’ from PPI contributors’ with specific, local concerns and one off projects to their developing broader and sustained lessons and collaborations [9], such that being part of a regional research programme like a CLAHRC might inculcate.

A second wave of CLAHRC funding (2014–2018) established thirteen CLAHRCs across England with three listing PPI as an explicit research theme. The current CLAHRC East of England (EoE) encompasses 36 organisations including those planning, commissioning and delivering health and social care within the region. Industry partners and three higher education institutions (HEIs) act as hubs across five research themes, three ‘stand-alone’: Dementia, Frailty End of Life Care, Enduring Disadvantage and Disabilities and Patient Safety; and two cross-cutting themes: Health Economics and Patient and Public Involvement in Research. Each theme was hosted by a HEI, an academic lead and had several funded projects. The PPI theme was unique in being both a research theme and having an operational role in advising and evaluating PPI within funding proposals and projects. CLAHRC EoE’s strategic principles for PPI were for it to be “embedded, comprehensive and active” in each project, and to be further developed over the CLAHRC’s duration.

- Recognising PPI as integral within every CLAHRC East of England project.
- Encouraging all CLAHRC projects to allocate minimum 5% of their resources to support PPI activity.
- Including diverse populations throughout the research from project design to implementation and dissemination.
- Encouraging links between PPI stakeholders and initiatives at local and regional levels to support active learning and collaboration.
- Identifying a PPI co-ordinator for each theme.

A PPI coordinating group reviewed grant applications against criteria for PPI and gave formative feedback. CLAHRC EoE also has ten Key Performance Indicators (KPI) and a KPI tool on which projects regularly report their activities. One KPI (see Box 1 below) explicitly relates to PPI:

**Box 1: Key performance indicator**

KPI 3. Engagement and involvement of service users, carers and the public in the research process from design to evaluation. (PPI) and in research programme governance.

CLAHRC East of England (EoE) PPI theme projects include its flagship ‘*IMPlementation and evaluation of Patient and Public Involvement (PPI) in CLAHRC East of England RESearch* (‘IMPRESS’), reported here. IMPRESS aims both to build evidence on whether PPI within a publicly-funded regional research programme can connect disciplines, partnerships and knowledge-building between PPI contributors and researchers [10, 11] but also can effect change and thus make improvements in research practice concerning PPI.

### Background to PPI

PPI in research is underpinned by range of epistemological, moral and consequentialist arguments. Boote *et al* page 45 [12] outline these arguments as follows: The epistemological argument posits health research as benefiting from the experiential knowledge and insights of patients, carers and service users [13]. The moralistic argument posits the right of the public to be involved in any publicly-funded research that may impact on their health or services they receive [14]. Finally, the consequentialist argument presents public involvement as helping improve the quality, relevance and impact of health research [15].

Recent systematic reviews of the evidence base, are widely cited as reporting the benefits of PPI in research as: identifying more patient-centred research topics; improving feasibility of study design; developing more effective recruitment strategies; more patient-centred data analysis; improved dissemination; improving researchers’ links to the wider community and in translational research [16–18]. Despite this recent growth in the PPI literature, [12] repeated calls have been made to assess PPI impact on research processes and outcomes more systematically [19, 20]. Reflecting such calls, the NIHR therefore commissioned three national studies of PPI impact in 2010 [21–23]. Of these three national studies, the realist evaluation by Wilson et al [23] ReseArch with Patient and Public invOlvement: a RealisT evaluation (RAPPORT), deployed Normalisation Process Theory (NPT) to examine how PPI becomes ‘normalised’ or embedded within research studies [24], (including some CLAHRC projects) and identified six ‘salient actions’ for effective PPI. These included: a shared understanding of moral and methodological purposes of PPI; a key individual co-ordinating PPI; ensuring diversity of PPI contributors; a research team positive about PPI input and fully engaged with it, based on relationships maintained over time; and PPI proactively and systematically evaluated.

The role of organisational contexts in supporting PPI is acknowledged as crucial, yet rarely analysed [25]. An evaluation of the first wave of the CLAHRCs [26], recommended future research examine ‘the key enablers of and barriers to successful patient and public engagement in research production and implementation in collaborative partnerships such as the CLAHRCs’(xxvii). Another evaluation of the first wave of CLAHRCs [27], reports publications exploring PPI in the CLAHRCs. CLAHRC NWL’s (North-West London) PPI publications relate to how patients’ views on PPI differ from those of healthcare professionals [28], and how a particular healthcare quality improvement initiative, (rather than research programme *per se*) explored how patients used elements of the new organisational culture created to collaborate with healthcare professionals [29]. This ‘new organisational culture’ was a distinct quality improvement initiative, where project teams of 8–10 multidisciplinary frontline NHS staff and patients (PPI), adopted specific quality improvement methods to plan and test small changes in care. The organisation held regular collaborative cross project shared training/learning events to develop this ethos iteratively. As the programme evolved CLAHRC NWL ‘increasingly emphasised PPI as a key means to achieve improvements in care’ (2015:22) and ‘patient participants positioned themselves as active and legitimate improvement agents, constructing their interpersonal relationships with healthcare professionals as equal and collaborative ‘ (2015: 31).

Jordan et al (2015)’s published research from CLAHRC NDL’s (Nottinghamshire, Derbyshire, and Lincolnshire) analysis of the research team–service user relationship from the service user perspective [30], demonstrates how the analysis of ‘The Three Rs’: roles, relations and responsibilities between researchers and service users may help ensure that patients’ expectations in relation to PPI match their actual experiences for healthcare research organisations. Structured via ‘The Three Rs’, aspects of the relationship are evaluated (e.g. motivation, altruism, satisfaction, transparency, scope, feedback, communication, time). As with our IMPRESS study, this paper is reporting findings from a programme of research projects, but informed by medical sociology, rather than being an action research project.

The IMPRESS study, reported here, explicitly builds on the NIHR-commissioned realist evaluations of PPI and reflects the RAPPORT study [23] (mentioned earlier) in also employing Normalisation Process Theory [24], to investigate how PPI may be embedded within the specific context of a regional research programme, namely CLAHRC EoE. IMPRESS goes further in employing action research methods to identify and inform prospective actions to implement, test and more fully embed PPI.

The IMPRESS study objectives were:

1. To explore the experience of PPI in research within a CLAHRC for both researchers and members of the public
2. To identify and address barriers and enablers to “fully embedded, active and comprehensive PPI” in a CLAHRC research programme
3. To determine what is entailed in “normalising” a programme of PPI across a CLAHRC, informed by stakeholders’ views and experiences of contributing to that programme

This paper describes the methods of IMPRESS and the work completed so far, to develop PPI action points to improve PPI within the CLAHRC EoE research programme and share its lessons.

## Methods

### Method of enquiry: Normalisation Process Theory as a preliminary-step in action research

An Action Research (AR) approach [31] balances problem-solving actions implemented in a collaborative context with research to understand underlying causes, to enable predictions about personal and organisational change. AR encourages diverse stakeholders to engage in cycles of four sequential stages: information collection, planning, action and evaluative review, which inform later cycles to test and refine earlier-developed actions and interpretations. This IMPRESS study follows an AR approach by Lewin [32] in including a substantial ‘preliminary-step’ of current state fact-finding, here of PPI within this research programme. Data collection and analysis were informed by Normalisation Process Theory (NPT). Findings are then used to plan, act, evaluate and refine information to develop further findings through subsequent AR cycles, delivering sequential programme- and project-level analysis of PPI implementation within a CLAHRC.

Normalisation Process Theory (NPT) is a theory developed for building working concepts (“middle range theory”) [24, 33, 34], to explore how innovations or interventions are implemented and ‘embedded’ in organisations.

NPT has four domains for examining processes of implementation:

- Coherence: making sense of the innovation as different to previous work
- Cognitive participation: buying-in to the work required for successful innovation
- Collective action: doing this work
- Reflexive monitoring: systematically evaluating the innovation and responsively modifying the work

Each construct has four sub-components for further sensitising concepts (see S2 Appendix).

In IMPRESS, NPT allows us to explore; how different research teams individually and collectively understand PPI in the CLAHRC; how far PPI is embedded in terms of whether and how research case study members make sense of PPI, buy into it, operationalise it, and appraise its effects.

Both heuristic devices (AR and NPT) are epistemologically compatible, both located within the social science constructivist paradigm, combining well in other studies of research translation and community involvement in primary healthcare [35]. They offer, in combination, ‘a more effective means of supporting implementation projects ‘and of providing deeper understandings of implementation contexts, rather than merely describing change’ [35]p20.

### Design

**Fig 1:**
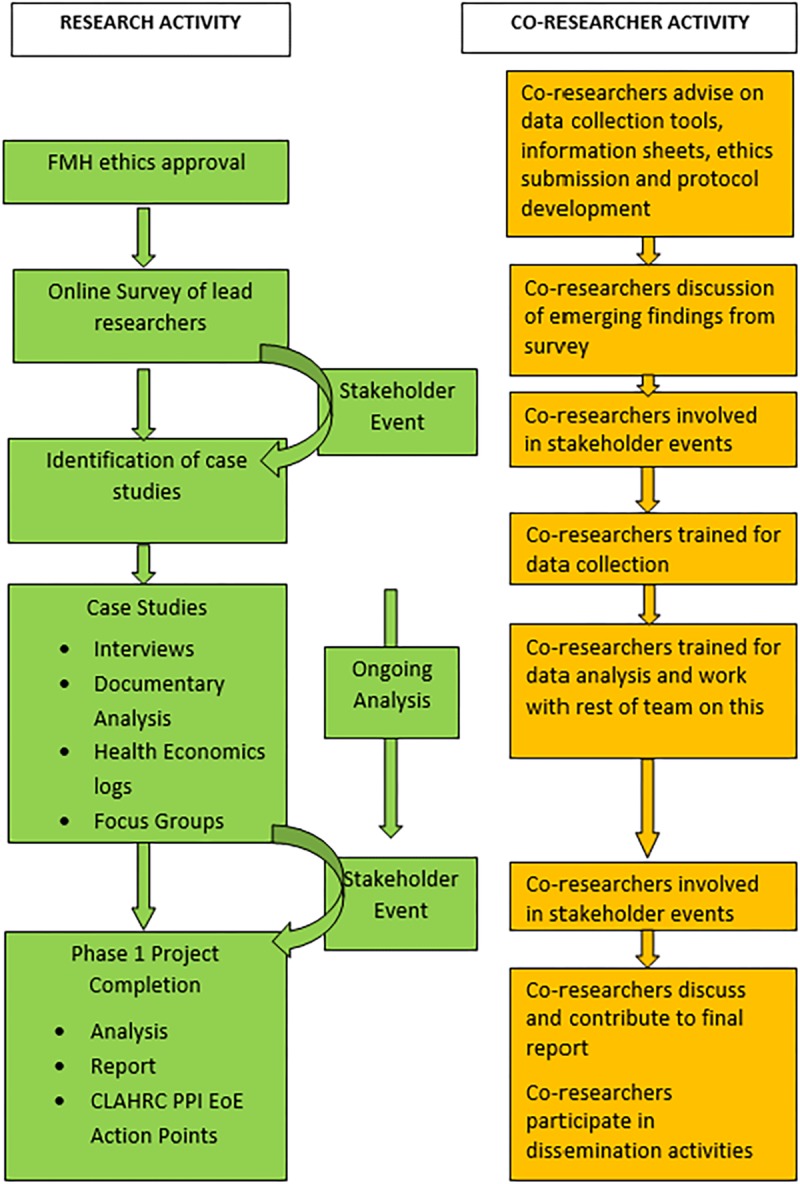
IMPRESS project flow chart.

We utilised a sequential, mixed-methods and case study design; and here report findings from the qualitative case study phase. The project flow chart (Fig 1) illustrates research phases, methods employed and PPI co-researcher collaboration.

### Ethics statement

The University of East Anglia’s Faculty of Medicine and Health’s Research Ethics Committee approved the study (Ref: 2014/2015 17) which invited participation from younger people, people with intellectual and developmental disorders. The team was skilled working sensitively with these groups, although (as noted in the findings section) relatively few could take part. We were also careful to remain neutral in our reactions to reports of practice, mindful and sensitive to potentially exposing bad PPI practice, while probing carefully to clarify understandings and explanations. Participants all received an information sheet detailing the purpose and ethical conduct of the research and signed a consent form.

### Recruitment and sampling of case study projects

A list of all the 27 projects funded by the regional CLAHRC, and their Principal Investigators’ (PIs’) contact details were obtained through CLAHRC EoE central office. All PIs were then invited to participate in an initial PPI survey (not reported here). Following receipt and analysis of questionnaire data, we devised a sampling frame to purposively select 8–10 case studies (each of a single research study) ensuring typicality and maximum variation across CLAHRC EoE research themes, research types (from systematic reviews to lab based studies, to qualitative studies), topics and PPI design (how many PPI contributors were involved, at which stages of the research, and the nature of their roles). We considered how cases represented the various CLAHRC institutions and partners, the current research stage reached of each and the study’s overall duration.

PIs of potential case study research projects were sent an IMPRESS study invitation and Project Information Sheet. Once agreed, projects forwarded interview invitations and information sheets to their researcher team including PPI contributors. Participation was voluntary and exclusion criteria included: those aged 13 years, or under, those unable to give informed consent, any PPI contributor who is also a current patient of (clinical) investigators on the research project and those unable to speak English. We recognised that specific strategies were needed to reinforce messages about the nature of informed consent and confidentiality for young people or persons with intellectual or developmental disorders, so an ‘easy read’ version of the information sheet was also developed. This included explanations of their right to not answer specific questions or to withdraw at any point, and we would agree a form of words or action they can use if they wished to stop the interview. For any participant aged 13–16 consent from parent/guardian would be sought, and for those with an intellectual or developmental disability advice about their ability to provide informed consent would be sought in advance from project leads, as they had already consented to take part in the case study project. Potential case study participants for subsequent focus group discussions were identified from those already interviewed and were sent further e-mail/post and relevant invitation and information sheets.

### Case study interviews and document analysis

Case studies were each allocated to an IMPRESS researcher: JK, AV, HW and JuB, all social scientists experienced and trained in interviewing, making field notes and analysing qualitative data. To maximise recruitment and participant convenience, one-off 30 minute telephone interviews were conducted. The option of face-to-face interviews was also open to participants and vulnerable interviewees were offered the option of having a non-participant present. The interviews were semi-structured and conversational, enabling informants to freely describe their experiences and raise unanticipated topics.

Two NPT-informed topic guides (for researchers and for PPI contributors, see S1 Appendix) were adapted (with IMPRESS PPI co-researchers) from those used in the RAPPORT study. Both topic guides covered the same core topics, tailored for different participants and iteratively, to reflect ongoing analysis within that case.

### Focus groups

Case study participants were invited to join separate researcher or PPI focus group(s) (up to ten people), to represent their case study in discussing interview analysis themes. Our two PPI co-researchers received training to support focus group planning, discussion guide refinement and focus group facilitator and observer roles. Focus groups were scheduled and tailored to suit participants’ confirmed preferences. Three one and half hour focus groups ran within working hours at institutional offices. Discussions were facilitated by a researcher JK or HW and co-researcher AW and observed by co-researcher PV, who took field notes. Interview and focus group recordings were transcribed verbatim for content, rather than sub-vocalisations and pause duration. Researchers checked transcriptions, field notes and documents for accuracy and anonymised them before entering this data into computer analytical software (NVivo).

### Stakeholder events

Two IMPRESS stakeholder events were held to engage CLAHRC members (including PPI contributors) and regional PPI groups. The first shared plans for IMPRESS, initial survey results and further scoped, refined and validated the IMPRESS study. Attendance for the second event was widened to include national representatives from other CLAHRCs and PPI organisations to share initial case study findings and discussed implications for evaluating and building stronger PPI within a research programme.

### Analysis

Case study data were analysed within- and across-case in a rigorous, ongoing process of continual re-reading related to the study’s objectives, exploring the presence/absence of new themes and posing further questions. An abductive analysis combined deductive reasoning informed by the NPT four domains and 16 constructs adapted to our research objectives and additional inductive, thematic coding (see S2 Appendix).

PPI co-researchers’ training included NPT introduction and they made valuable contributions to analysis, adding standpoints and insights to deepen interpretation and ‘member check’ the representativeness of the final analysis across fully the breadth and depth of accounts collected.

Our analysis reflected the RAPPORT study approach [23] and included the following stages:

1. A subset of initial interview transcripts were coded independently (manually) by team members (JK, AV, HW) and trained PPI co-researchers (AW and PV), guided by NPT- deductive analysis and inductive analysis of emergent themes relating to our research questions [36].
2. A coding meeting was held to discuss the processes, identify and collectively address inconsistencies in applying coding categories, to refine collective understanding and to identify deviant cases of category absence or presence.
3. All data relating to the identified categories were highlighted within each case study by (JK, HW & AV), using NVivo 9 software (QSR International, Warrington, UK). Coding decisions were annotated (within NVivo), discussed and revised at regular team analysis meetings before finalising a coding frame (see S2 Appendix).
4. Data were triangulated within cases [37] and case summaries written and shared within the team to check consistency, degree of collective implementation within case studies, and to identify data limitations and gaps in our understanding.
5. The web-based NPT toolkit [33] was then used to develop radar plots for each case study to visually represent PPI embeddedness within a case study in terms of NPT constructs and domains.
6. Following initial analysis by case, independent, duplicate analysis was undertaken for each theme across cases (by one academic lead FP, JB, AW and one researcher JK, HW, AV per node) and discussed at team meetings. PPI co-researchers agreed to focus on two specific key themes with a slightly different emphasis, to consider more widely what the data indicated for the main IMPRESS research questions and to consider any key omissions. The across-case node reports produced headline findings, questions for further analysis and notes for potential PPI action points for the CLAHRC.
7. Research themes and findings were then shared and debated with regional and national stakeholders to further refine them, and produce final node reports.
8. A final list of ten PPI action points was then co-devised amongst the research team.

In presenting findings we set out and evidence our interpretations by providing illustrative data extracts for each interpretive point. We indicate how each extract is drawn from our dataset and can be contextualised in specific cases (listed in Table 1) by presenting each extract identified with an extract code. These codes provide for each extract the identification number for the related case study, and the participant number group (PPI or Res) of the participant or participant group being quoted. Thus CS2Res02 is Researcher02 interviewed from Case Study 2.

### PPI in IMPRESS

PPI was embedded in all IMPRESS project stages from design through to producing its written outputs. IMPRESS involved two PPI co-researchers (who were PPI advisory members for RAPPORT), a lay chair for its project advisory group, and three regional PPI contributors recruited via an application process following advertisement. Co-researchers were involved (in line with their ongoing preferences and support needs) in conducting focus groups, stakeholder events and qualitative analysis, writing up and disseminating findings for various stakeholders/audiences. They received support and training in NPT, the conduct and analysis of focus groups and are acknowledged (as co-authors or otherwise) in all outputs. Honoraria were paid and costings budgeted for, following INVOLVE guidelines, comprising 5% of the IMPRESS budget.

## Findings

### Sample

We recruited 10 out of 27 active CLAHRC EoE projects as case studies to IMPRESS. Of the original 10 projects invited to participate, eight accepted and two declined (one saw taking part as too burdensome given their other commitments, and another declined to acknowledge invitations). Two more studies were then invited and accepted. The crosscutting health economics research theme, having inter-related research teams, mostly performing secondary analysis on trial data sets and none with active PPI, were treated as one case study.

For six case studies we managed to recruit both researchers and PPI contributors. For the other four, two had no PPI contributors at this time and two had PPI contributors not possible to invite as they did not meet our ethics inclusion criteria. In total, across ten case studies, 24 researchers were interviewed, and 13 PPI contributors, from June to November 2015. Five researchers and four PPI contributors were interviewed over the phone. 19 researchers and four PPI contributors were interviewed face-to-face. Four PPI contributors (adults with learning difficulties) were interviewed with a case study researcher known to them for support.

The response rate for researchers was 24/30 (80%). In some cases, research staff were no longer employed on the project or did not respond to invitations. It is not possible to give accurate figures for PPI collaborator response rates, because not all projects knew how many PPI contributors had been involved on projects, where projects had worked with regional PPI groups who collate anonymised feedback. Also, as numbers or contact details for such PPI contributors invited were not available, we cannot say how many declined or why.

The 10 IMPRESS case studies and their PPI are detailed in Table 1 below. They ranged in length: 12–36 months, with the majority employing mixed methods designs. Two PPI models were identified, and classified in line with RAPPORT study findings [23]. Firstly, a ‘One-off’ PPI model where PPI collaborators undertook a limited researcher-identified task with or without outreach: where PPI collaborators bridged the research and a wider community. Secondly, a ‘Fully intertwined’ PPI model where PPI collaborators worked alongside researchers throughout the research process.

**Table 1:** IMPRESS case studies and their organisation of PPI.

Case Study	Type of research	PPI model, PPI roles	PPI forums	Interview
1	Mixed methods: evaluation	**Intertwined PPI with outreach** 4 x PPI (1 x Advisory group; 1 x Advisory Group + Co-researcher; 1 x national patient rep; 1 x Hospital advocacy officer)	Advisory group+ Co-researcher	Res = 2 PPI = 2
2	Mixed-methods: service mapping and health economic costing	**Intertwined PPI** 8 x PPI recruited for previous linked study.	Advisory group	Res = 5 PPI = 5
3	Mixed methods: physiological monitoring and qualitative	**One off PPI** 9 x PPI recruited through a local patient day centre	PPI day- pilot work	Res = 1 PPI = 0
4	Mixed methods. Participatory Research	**Intertwined PPI** 9 x PPI Duel role (both research participants and co-researchers). Steering group includes 1 x parent/carer collaborators	Co-researchers Steering group	Res = 2 PPI = 0
5	Secondary data analysis	**One-off PPI** 2 x projects in theme consulted PPI panel	Telephone consultations and meetings with service users regarding patient pathways and design and timings of questionnaires	Res = 2 PPI = 0
6	Mixed methods: exploratory design and systematic review	**Intertwined PPI with outreach** PPI from previous study fed into research questions. Wide consultation by CI of local SU groups. 4 x PPI on current project + 1 x PPI co-researcher piloted the data extraction tool	PPI from 2 previous projects fed into CS06. Face-to-face consult with local SU charities/orgs etc.; Project steering group stakeholder group	Res = 2 PPI = 2
7	Systematic Review	**One-off** PPI group (of SU) set up for previous study consulted in development of this study 2x local PPI groups (general PPI)	Virtual, via e-mail/post with one PPI group. Face-to-face with another PPI group. Comment on protocol and suggest questions for literature review	Res = 4 PPI = 1
8	Mixed Methods: document analysis, observations and systematic review.	None		Res = 2 PPI = 1
9	Qualitative: Evaluation	**One off** The project has had difficulty with securing a clear direction and has struggled with defining and implementing PPI. (Carer Collaborator)	PPI contributors and other stakeholder reviewed project proposal; advised on the advertising of the project to service users and Collaborator) with the writing of a lay summary	Res = 2 PPI = 1
10	Mixed methods	**Intertwined** PPI group (generic)	Advisory group also advising on documents and processes.	Res = 2 PPI = 1

Abbreviations: Res = Researcher, PPI = Patient and Public Involvement contributor, SU = Service User, CI = Chief Investigator.

PPI models related to the academic researcher’s discipline, the study aims, the quality of established PPI relationships and study time and resource constraints. The PPI roles ranged from one-off, up-front consultative events to collaborators acting in a consultative capacity on an advisory group, to those acting as PPI co-researchers.

Case studies forwarded a total of 28 supporting documents to our IMPRESS study team, including: study protocols, meeting minutes, PPI consultation responses, researcher’s responses to PPI contributors, posters, PPI training materials. Three focus groups were held. Two with case study researchers (n = 4 and n = 6 respectively) and one with case study PPI contributors (n = 5). Two stakeholder events were also held with 23 and 24 attendees respectively.

**Fig 2:**
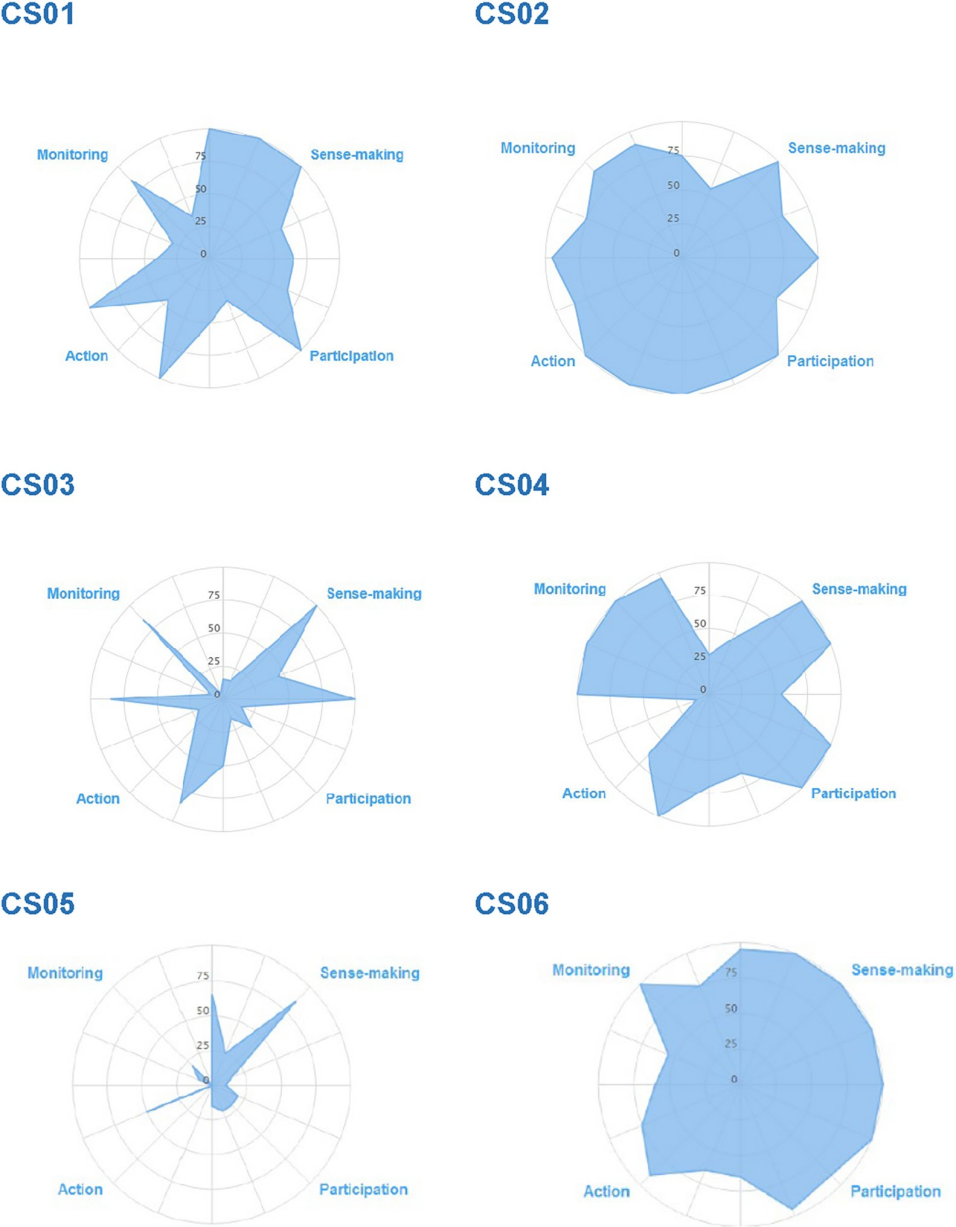
IMPRESS Case study NPT radar plots.

NPT radar plots for case studies (see Fig 2) show the embeddedness of PPI within projects across the four NPT domains (quadrants) and subcomponents. The fuller the radar plots, the more fully embedded the PPI. Spikier plots illustrate internal variance in degree of embeddedness. Acknowledging gaps in data collected, these plots were used as useful heuristic sketches to inform our interrogation of data and invite stakeholder interest. They are reproduced here with caution as only representing the data and interpretation available at one time in a project’s research cycle, and the expression of embeddedness will partly reflect the PPI design on different projects e.g. a project with little PPI would not be expected to have developed means of appraising its effectiveness. Case studies’ NPT radar plots are further neither simply or directly comparable as projects/PPI cannot be defined as closed entities, e.g. some following from a previous CLAHRC funded study and previously established relationships, others forming component parts of a larger study as with the Health Economics theme.

This section now details findings on PPI embeddedness within case studies and the programme across the four main NPT constructs. Material points are summarised at the end of each section.

### ‘Making sense’ of PPI

Findings here relate to the work that research teams do when making sense of PPI and to what extent PPI is an internalised way of working.

It was the case study lead academic or Principal Investigator (PI) who held the broader overview of their project’s PPI, with contract research staff managing projects often less clear about rationales for PPI, and what PPI activity preceded their employment.

Those PIs more experienced and confident in PPI, asserted that universal definitions of PPI design, roles and purposes, cannot be taken for granted and are perhaps not desirable, see also [38].

*‘* …*it [PPI] cannot have a definitive definition even*, *so the important thing when you’re developing anything to do with your research is to just make it very clear about [what] you think it is as a researcher and have those clear definitions within your own research* (CS01Res01).

Indeed, the range of definitions given, which underpinned project-specific PPI roles, resulted in critiques (as below) of more ‘operational’ NIHR-type definitions of PPI, which resulted in fixed and formulaic processes of PPI:

*‘…it [PPI] becomes an ingredient of your research …and then there becomes a whole raft of ways of understanding the quality of that*, *some of which I think are important and good and some of which I find frustrating and steer it towards a more what I would think of as formulaic activity’* (CS07Res02).

The confidence expressed by researchers in Focus Group 2, in doing the work of defining (or co-defining) PPI in context, contrasts with the nervousness of Focus Group 1 researchers, seeing such tailoring of PPI as potentially problematic. In contrast PPI had to be meaningful and non-superficial, requiring more guidance and evidence-based practice, see also [39]:

*Res02: I do have that feeling that there probably is a better way or a correct way of doing it, and that I’m just kind of making, I feel like I’m making it up as I go along*.

Facilitator: Do other people feel like that?

*Res03: Yeah*.

*Res01: Yes*.

*Res03: Definitely*.

*‘…what is a kind of good standard to be aiming for*, *what is the gold standard*, *how do we interpret that in the different settings’* (CS02Res03).

The expertise required to align PPI best practice research and theory within CLAHRC context is present within CLAHRCs own PPI theme (and accessed by some case studies). Some researchers felt that CLAHRC contexts (requiring much less detailed funding applications than other, larger NIHR awards) were open enough to support ideals of negotiating PPI roles in a flexible, organic way. However, they also recognised that being flexible with PPI could be difficult in the context of delivering CLAHRC research projects of shorter duration:

*‘…you tend to revert to type*, *particularly if you’re under things like pressure and you’re thinking*, *I know there’s a group there that I can access*, *or you now*, *a lot of the already support groups*, *networks*, *they know what to do*, *you’re there*, *but it does… take away the potential to be imaginative’* (Res04 FG2).

Linked to the dangers of PPI becoming too formulaic, as it is embedded [38], is another of that drawing upon convenient, pre-formed, pre-trained, supported, generic PPI groups raises questions for the diversity and representativeness of the PPI contributors within CLAHRC EoE, as expressed in the quotation below, see also [40–42].

*‘Yeah*, *well it’s just very easy isn’t it because we just have quick access to people…It never occurred to me to look outside the [local group 1]’* (Res03 FG2).

Such efficiencies of working with established PPI groups have implications for networking beyond those already engaged in research and developing a programme-wide PPI culture, and wider social justice concerns about who is represented and excluded, see also [43]. Lack of run-in time both with developing research applications and setting up projects also disadvantages teams with little familiarity with, or responsibility for, developing PPI, particularly for researchers not networked with existing groups and expertise:

*‘…you know*, *we’re not a medical department*, *so …generally what we do in my division*, *my group*, *is quite unique to the department*, *so it’s very unlikely you get institutional*, *significant institutional support [for PPI]’*. (CS08Res01).

Finally, another key finding in how case studies made sense of PPI, was that the different persons interviewed (senior academics, practitioners, contract researchers, PPI contributors) varied in knowledge of the CLAHRC as a distinct programme and a funder with specific commitments to PPI, only expressed by one senior researcher:

‘*CLAHRC projects have to be implementable don’t they in some way so we’re expecting the lay people that we actively involve to help us with the implementation side of things as well as the research’* (Res01 FG2).

This lack of differentiation with respect to CLAHRC commitment to PPI was certainly the case for PPI contributors interviewed for IMPRESS, especially where PPI contributors were already members of established PPI groups, primarily involved in one-off consultations and recruited to multiple, short-term projects within but also beyond the CLAHRC.

PPI contributors interviewed therefore often talked about their broader involvement in research and struggled to distinguish how they were involved in any specific CLAHRC project, or in recalling any specific project activities. They had little awareness of the CLAHRC as project funder or feeling of being part of a wider research programme with any specific identity or approach to PPI. This was also the case for some researchers and academics recently joining the CLAHRC:

*‘even the communication with CLAHRC was not very clear at the beginning so we didn’t get really to feel being a part of CLAHRC until it was nearly time to leave it’* (CS07Res03).

In summary, our findings show that:

- Despite this CLAHRC’s commitment to PPI in research, there was marked variation between case study projects, institutions and research themes in relation to their baseline knowledge and experience of PPI in research.
- There were few reported mechanisms or opportunities (time/space) to support programme-wide PPI learning.
- Nervousness was reported in awareness of and deviating from ideals of ‘best practice’ PPI. Concerns were also expressed about PPI becoming too formulaic and that under pressure research teams reverted to ‘tried and tested’, familiar PPI models of PPI rather than looking to innovate or to question established approaches.
- Most felt that the CLAHRC lacked an identity in practice and that its distinct commitments to PPI were not widely known.

### ‘Buying in’ to PPI

The second NPT construct refers to the ‘buy in’ work that people do to build and sustain a community of practice, here around PPI in research.

Buying into PPI was harder for researchers in themes ‘a step-removed’ from patients and service users. Researchers in the Patient Safety theme had fewer pre-existing PPI relationships and contacts with PPI organisations; and whilst the Health Economics theme was more familiar with PPI in research, their work is more often a component of a larger healthcare research trial, with PPI implemented at a wider level:

*‘…that would be more the [trial] project manager that was going to be doing it [PPI] or one of like the researchers that are doing the day-to-day trial’* (CS05Res02).

Those themes closer to patients and service users could draw on existing PPI contacts. For example, clinicians and academics within regional service user networks can keep research and PPI in view between funding calls, particularly useful for bid development when there is a local funder:

*‘…where CLAHRC does have a particular something to bring*, *is that it’s slightly different from the purely project stop*, *project stop*, *project stop*, *kind of other sources of funding which make it very difficult for those relationships to develop where people who research isn’t their first business’* (CS07Res02).

Working with familiar individual service users or PPI groups with whom relationships are already established was viewed as efficient and pragmatic, particularly if PPI was a one-off consultation. Individual PPI contributors could also be re-involved from prior projects (funded by CLAHRC or otherwise). However, prior relationships could potentially block, as well as facilitate PPI, as some experienced PPI group members interviewed reported being selective about working with researchers perceived to be ‘paying lip service’ to PPI in research. More broadly, the time pressures surrounding new CLAHRC projects actually *relies* heavily upon the ‘industry structure’ of established PPI groups and service user networks saving the costs of training, support and administration:

*‘If you’re going to find new members of the public to be involved*.*…are you still providing the kind of support and training that they need*? *And I know that*, *you know*, *with the [PPI group]*, *we have the industry structure there to provide that support’* (CS10Res02).

But knowledge of and relationships with existing PPI and service user groups was not equally distributed or shared across the CLAHRC research themes or case studies with some researchers reporting a lack of wider CLAHRC networking around PPI, leading to feelings of isolation:

*‘…we’re all chums together but we don’t actually help each other*, *we’re sort of passengers sailing in the same ship*, *but we have our own berths to sleep in…’* (CS09Res01).

These researchers later benefitted from support provided by the PPI theme (once set up) in advising on PPI design and signposting to possible sources of recruitment of PPI contributors. Beyond initial applications and project set up periods, dealing with the day-to-day operational or administrative aspects of PPI requires inter-personal skills, see also [23]p124-125. Such aspects, e.g. supporting individuals’ requirements for diet, access and processing expenses claims etc., were often dealt with outside project teams, by co-ordinators within PPI organisations. However, where dealt with internally within project teams, such aspects were delegated to junior members of research teams yet often the required sensitivities not clearly defined or formally recognised within job descriptions.

CS2’s PPI collaborators were vulnerable adults recruited as a bespoke group and all new to PPI in research. This fully entwined PPI model entailed substantial set up and ongoing costs, with most research team members (whatever their position) engaged in providing ‘one-to-one’ buddying or practical support to get PPI contributors across the region to meetings. Some researchers were thus conscious of the ‘opportunity costs’ in allocating roles for operationalising their PPI to senior staff:

*‘He’s (the grant holder) happy for (name of researcher) to do it*, *he’s very in favour of PPI*, *but he thinks (name of researcher) should be doing it*, *and people like me should be writing papers’* (CS02Res05).

Indeed, an economic evaluation of their study PPI model raised team doubts over its legitimacy. Whilst clinicians endorsing this model entailing extensive support as integral to the study, others questioned the value of this investment.

More widely than this case study, researchers interviewed for IMPRESS broadly accepted PPI as part of contemporary research practice. Reasons given for PPI’s legitimacy rehearsed the rationales reported within the policy sphere and PPI research evidence: epistemological, moral and consequentialist (reviewed above) The *Dementia*, *Frailty and End of Life Care* and *Enduring Disadvantage and Disability* research themes prioritised the moral and epistemological rationale, others e.g. *Health Economics*, the consequentialist. Many researchers also, however, questioned the legitimacy of PPI in CLAHRC projects with respect to the relatively small grant size but also limited time to do PPI ‘properly’, aligned with best-practice guidance.

PPI contributors spoke in general terms about the legitimacy of PPI, and the unique contribution they could make to projects as distinct to that of the researchers:

*‘…we come up with all sorts of things that the researchers do not think of*, *and we give them a hell of a lot of material …food for thought’* (CS07PPI01).

The PPI contributors’ focus group endorsed the need for PPI on all projects (as specified by this CLAHRC), questioning the constraints set on PPI within projects when restricted to advisory group membership.

Finally here, PPI contributors reported that projects did not always keep PPI ‘in view’, being asked to do *‘isolated bits on isolated bits of paper*’ (CS01PPI01) suggesting that researchers find better ways to keep them updated about projects, especially as many are active members of multiple PPI organisations, involved in multiple projects:

‘…*just a few lines just to keep it fresh in your memory*, *you know*, *because you’ve invested your time into it and you don’t want to lose that investment’* (CS06PPI02).

Researchers in turn acknowledged the importance and challenges they faced in keeping PPI in view, whilst also progressing the research.

*‘You’re often juggling various different projects […]*, *just trying to make sure things are running to time and all those kinds of challenges*. *So it [PPI] can be that sometimes*, *you know*, *it’s something that perhaps slips*. *It’s not that you don’t think it’s important*, *but you know*, *maybe it does slip down the list of priorities’* (CS10Res02).

Those case studies that involved PPI contributors on a regular basis had committed to tailor their ways of working to maintain communication, as discussed next. Other researchers reported difficulties maintaining PPI contributors’ buy-in when struggling to maintain contact between meetings, when PPI contributors had to miss meetings or drop out due to ill health, or when PPI contributors were quiet at meetings or seemingly not engaged with tasks assigned to them in ways anticipated. In summary, our findings demonstrate:

- Not all projects or team members automatically ‘bought in’ to or set up PPI, particularly those less directly involved in patient care.
- Cross project, intra-theme support for PPI within the programme was also lacking, and in the initial stages of this CLAHRC the PPI theme’s operational role was under development.
- After recruiting PPI contributors, those projects enacting a more intertwined PPI model were better set up to keep PPI in view than those where PPI roles were one-off consultations.
- Critical engagement with PPI was evident within our data, especially around issues of PPI legitimacy. Such ongoing debate within projects as to why they were doing PPI and how they have set up to do it, is actually a key first step towards adapting PPI.

### ‘Doing’ PPI

This construct refers to the operational work in implementing PPI, including institutional protocols, policies and PPI-related procedures in the CLAHRC and organisations that host PPI groups. Again, we found extensive benefits, cost- and time-savings from working with established PPI groups which had funded co-ordinators.

‘…*you didn’t have to think about the relationship side of PPI too much because that was kind of managed by the (PPI organisation 1)’* (CS07Res03).

These PPI co-ordinators were often on first name terms with PPI contributors, credited with abilities to: ‘*look after the nitty gritty’* (CS01PPI01) and *‘unblock blockages’* (CS05Res05). Unfilled or over-stretched co-ordinator posts could often lead to less-sustained PPI. PPI integrated in institutions which hosted projects and PPI groups was seen to encourage researcher-PPI collaborator communications about funding decisions, feedback on PPI contributions, ongoing project updates, robust rapport and common understandings. The regional PPI groups train their volunteers, with projects offering additional training for PPI contributors undertaking co-researcher roles such as data analysis (CS1) or specific tasks (CS4). Having shared expectations around communication was recognised as important:

*‘I spend a lot of time communicating with PPI people on the project*, *which couldn’t be quantifiable and stuck in a form back to the CLAHRC or whatever*, *but it is a lot of phone calls or emails and that’s really important …that you just keep that contact going …‘cos you know they’re busy on other projects*, *so it’s a balancing act trying to get the communication right*, *but I think it also helps if you know what the PPI member is expecting on the project in terms of your communication for their involvement […] right at the beginning*. (Res03 FG2).

Projects which set up their own bespoke PPI groups (CS2) or events (CS3) did so utilising their clinical understandings of the client group. So CS2 worked with PPI contributors to develop training to help members understand research, their role and the study purpose.

*‘* … *none had the ability to sit through a two and a half hour meeting and concentrate the whole way through*, *or …meaningfully contribute*, *so we asked them what they liked*, *what they didn’t like’* (CS02Res01).

The PPI contributors interviewed for CS2 expressed how they felt their contribution was being recognised:

‘*People [researchers] they’ll listen*, *just talk to them*, *we’re not funny people*, *just different people helping*, *with different things to talk about’* (CS02PPI04).

No specific training was given by CS3, but the researcher framed and focused the involvement task and process for them. Thought given to making PPI accessible was also evident in organising meetings at convenient times (e.g. for the young people in CS4 to fit in with educational commitments).

Our findings also demonstrated the importance of reviewing the allocation of PPI work to check its appropriateness to skill and comfort levels of PPI contributors, and to offer them appropriate training, and support. Twice researchers described over-estimating PPI contributors’ confidence levels to perform certain tasks; assuming this meant they could take on a more public-facing role:

*‘…we thought well okay*, *can we pare it right back and just bring it back to its sort of bare bones of the toolkit and I think they were more confident to do that*, *but not the actual workshop…’* (FG1 Res01).

Whilst CLAHRC funding processes approved plans for PPI in research, plans still needed to be adapted to circumstances. Whilst researchers recognised this, any consequent changes were likely under-reported as they were not formally documented or monitored by funders, see also [23]p114.

Research has highlighted the importance of mutual understanding and trust in developing relationships to ensure successful PPI [18, 21, 23, 44]. Relationships are especially important for projects where PPI is extended over time, beyond one-off events. In recognition of this, creative, fun facilitation techniques were sometimes used to help maintain relationships:

*‘…so people could learn about research but in an enjoyable way so it wasn’t seen as all work’* (CS02Res04).

Making time to actively listen to service users’ wider concerns and experiences was deemed important in building trust especially when working with vulnerable or marginalised groups, and in some cases PPI meant researchers relinquishing control of some project decisions:

‘*So this girl (PPI contributor) designed the poster with me which was difficult for me because I knew what I wanted*! *So that’s the key thing*, *[…] it means*, *a lot of compromise*. *But actually the poster turned out really well and she came to the conference*, *stood by the poster and answered questions so that was great for her’* (Res02 FG1).

These instances of experienced mutual acknowledgment contribute to building trust within the local context, upon which future projects can draw.

Senior researchers felt they had budgeted appropriately for PPI, and knew about the CLAHRC’s minimum 5% resourcing guidance. Most contracted researchers were vague about budgets, including PPI budgeting, and whether it was adequate. One focus group noted the lack of CLAHRC funding for PPI in developing projects, which also limits potential for having PPI-led projects or PPI as co-applicants:

*‘…there’s just no funding for that pre-work is there*, *and it takes time*, *building up relationships and trust […]that takes time to work with people doesn’t it*, *to suss that out’* (FG2 Res02).

They suggested a discretionary PPI fund for setting up new PPI in developing projects. CLAHRC-level support for PPI-researcher relationships between projects could particularly benefit ‘hard to reach’ groups, who cannot easily join generic PPI groups:

‘*I’m not convinced that the kind of engagement that we need to get from service users [PPI] is necessarily easily funded within these kind of two year*, *smaller CLAHRC mini-project ideas’* (CS02Res03).

Overall, researchers saw the CLAHRC as a bureaucratic funder which lacked PPI-specific processes (e.g. in research implementation) and communication to foster a sense of programme-wide PPI community:

‘…*considering the amount of [monitoring] information that’s asked of me I really don’t have any clear idea about what their vision is in relation to PPI and if it’s any different to anybody else’s* …’(CS01Res01).

Instead, sharing PPI experiences and guidance largely remained within-workplaces through formal presentations and informal discussion. In summary:

- Our findings detail the work done to put PPI into action in ways that recognise PPI contributors’ needs, so nurturing trusting relationships to ensure PPI processes which fit local purposes.
- Access to existing PPI groups with skilled, experienced coordinators can provide sustained relations for expediting PPI in newer projects.
- CLAHRC policies and guidance on PPI funding were acknowledged as supportive but bureaucratic, affording few opportunities to expand and sustain a regional CLAHRC-level PPI community. Discussions with the PPI theme’s researchers were seen as helpful, but not as delivering a wider PPI contributor pool for researchers to engage with for future studies.
- Where PPI contributors were suitable for specific projects but not also in existing PPI groups, or PPI coordinators lacked bridging skills, such as training to tailor project-PPI relationships, active PPI involvement was limited.

### ‘Appraising’ PPI

This construct refers to work assessing the effectiveness of working with PPI, methods and systems for appraising and reconfiguring PPI.

The CLAHRC EoE Key Performance Indicator (KPI) tool facilitates systematic reporting against the PPI KPI within projects. However, only some researchers and no PPI contributors used the tool for reporting or reflecting on PPI. Researchers expressed generally negative views of CLAHRC reporting mechanisms:

*‘I’ve not found the documentation for CLAHRC useful in any respect*, *I found it overbearing*, *unhelpful and time consuming*, *it takes my time away from what I’m supposed to be doing’* (CS01Res01).

*‘…it’s [PPI reporting] all about reports and KPIs and it’s just so tedious*, *and kind of drains the very soul out of me’* (FG2 Res04)

Those researchers who used the tool, suggested it was a useful log but discouraged holistic, nuanced communal appraisal by a focus on reporting positive impacts.

Some researchers’ expressed willingness to appraise, and to reflect on PPI in their own and others’ studies.

*‘…somebody needs to be thinking how am I going to measure on my project the impact of PPI […] but we don’t do that […]very well at all at the moment’* (FG2 Res04).

Researchers often saw evaluating PPI as a burden rather than a means for ensuring better PPI-project fit. Some researchers saw the CLAHRC’s role as providing advice on how to evaluate their PPI, regretting a lack of cross-theme appraisal of PPI and some suggesting a CLAHRC-developed PPI ‘standardised impact measurement’ tool, to record what PPI impacts were made.

PPI contributors, especially those with cognitive impairments, could struggle, without support, to appraise their project role. Indeed, several interviewees underlined the methodological complexities in formally evaluating PPI and defining and measuring PPI impact.

*‘[Sighs] No*, *you can’t evaluate that sort of thing*. *[Laughter] Well not in the controlled manner that …we’d ever tell whether it’s going to make a difference’* (CS05 Res01).

PPI costing and economic impact evaluation was found especially challenging. CS2 undertook an economic evaluation of PPI, later applying findings to reconfigure their PPI. CS4 had also formally evaluated young people’s involvement in their project through questionnaires, but no other case study projects had set up formal systems to routinely appraise PPI. Informal PPI appraisal was reported upon projects’ completion, often feeding into another research bid, or as a one-off review event, or sometimes as discussions minuted from team meetings, as this PPI contributor noted:

‘*In the actual minutes about the contribution I think that’s always*, *you know*, *nice to have your name recognised as having made that contribution’* (CS01PPI02).

We asked both researchers and PPI contributors to identify PPI impacts on the project, personally, or on PPI. Some projects had little PPI activity to appraise when interviewed (CS5, 8 &9). Many researchers struggled to articulate specific PPI impacts as actions rather than epistemological and experiential benefits:

“*… I suppose there were particular insights that they gave us… about the reality of their lives”* (CS02Res01).

Researchers also identified PPI-influenced changes to data collection methods ‘questions asked‘ (CS5) design, anticipating data collection issues (CS3) data extraction (CS7) and newsletter accessibility.

*‘…when they [PPI contributors] were asked hypothetically to wear that [monitoring device] in our project*, *they all said no[…]and it was the same pattern we found with participants that we recruited in our study*, *they were all happy to wear the chest device and the watch devices*, *but not the EEG*. *But the PPI is a great way to kind of*, *I guess*, *pre-empt that’* (CS03Res01).

Involving PPI in a literature review process led one researcher to note:

*‘I remember in particular how helpful those groups were in thinking about what are going to be the important outcomes we should be looking for in other people’s research*. *…I think that they both shaped up the topic but also*, *in most detail…the outcomes that we were looking for’* (CS07Res02).

Examples of communal team appraisal encouraging subsequent changes to PPI occurred most often when things did not go to plan or team members’ opinions differed strongly about how to carry out PPI (CS2) as with the earlier example of PPI contributors’ discomfort with leading events (CS6 & CS9).

Researchers also described PPI impacts on themselves, such as having their identities as clinicians challenged described as opening a different kind of relationship with service users in sharing personal issues.

*‘…wearing a different hat’s useful*, *hearing people in a group is very useful […] but it’s helped me work with my academic researcher colleagues in finding out […] the level of their unfamiliarity with people with learning disabilities*, *that’s been very helpful’* (CS02Res05).

The CLAHRC was seen as having potential for capacity building, shifting academics to more positive views of PPI, lessons for future projects, building collaboration and co-production. Two PPI interviewees identified a lack of mechanisms for developing user-led research in CLAHRC.

*‘…if there’s also the opportunity for the public to come along and say*, *this has been my experience of going through the cancer chain and it fell down here*, *[…]there’s got to be a better way*, *can you go away and find out what that better way could be’* (PPI101 FG).

Members of all projects identified potential for increasing (none for decreasing) PPI involvement in their projects and widening PPI groups’ membership to include ‘harder to reach’ PPI. Interviewees identified restrictions on PPI often as due to projects’ lack of duration or resources:

*‘We obviously always wanted to do more*, *but with the funding*, *and the time limit*, *and the nature of the systematic review*, *it was what we were able to do within these limits’* (CS07Res03).

The main negative cited by PPI contributors was often not knowing what their impact on the project had been. Several stressed the importance of receiving feedback from funding application stage through to dissemination and future project ideas.

*‘…it makes me wonder how I know that my contribution is of any purpose’* (CS10PPI01).

A CS6 researcher provided summary feedback following meetings, asking for comments on accuracy, making required corrections. In two projects, researchers reported sending feedback but which the PPI contributors did not recall receiving. Giving feedback can also require diplomacy, as when explaining why research cannot be altered as with validated questionnaires, or methodological approaches:

*‘I know we’ve had response rates that have been quite low and the PPI people in (PPI organisation 1) have said “can you go and knock on the door to get them to fill the questionnaire in*?*” […] they’re quite serious […] and you don’t know where to start in saying* (CS05Res01).

PPI contributors valued feedback detailing their individual contributions and their impact on the project, even if only at a one off event. They saw feedback quality as variable and not specific enough, while one PPI contributor acknowledged researchers’ busy schedule and so giving feedback lesser importance:

*‘Once they*, *have […] what they want from us*, *they concentrate on what they have to do*, *and …I don’t have a problem with that because*, *you know*, *they’ve got a job to do’* (CS07PPI02).

PPI contributors appraised their experience of projects most positively in terms of sociability and meaningfulness if involved throughout a project, had built relationships with researchers, or if they had learned something new. CS2 PPI contributors, with cognitive impairments found the being an advisor and belonging to a group, transformative:

*‘…people were asking ME questions*, *I thought I’m honoured’* (CS02PPI04).

But one PPI contributor found this transformative aspect tempered by some worry about the content of their discussions after meetings and indications that others would be upset when the research project ended:

*‘I can’t turn off*, *I have to think about it*, *over and over again’* (CS02PPI4).

The researchers were aware of this in managing this project’s conclusion. Another project raised concerns that PPI membership might constrain moving on from health problems.

*‘I have a worry that that participation for some young people might become a crutch rather than a kind of leg up to something else if […] for some young people their illness has become their identity’* (CS04Res01).

In summary:

- As the CLAHRC programme emphasised establishing rather than monitoring, ongoing learning or PPI process improvement, this may have tacitly discouraged projects from prioritising feedback to PPI contributors.
- Where formal appraisal mechanisms addressed the quality of PPI experience and contributions, these were seen as oriented more towards external institutional reporting of “what went well” and so less trusted when more critical insights were needed to develop within-programme PPI capacity and knowledge. However, some PPI populations could also require extra support, diplomacy and adequate explanation, to take part in such critical knowledge-building through mutual feedback processes.
- Attention was rarely given to specifying positive or negative PPI impacts on research outcomes. No ready-made tools were seen to review PPI input, but neither was there support for developing such tools in standardised “superficial” rather than co-designed, tailored formats.
- Communal appraisal of PPI was mutually appreciated rather than contested where occurring over longer periods within more developed, trusted relationships.

### PPI action framework

The PPI action framework (Table 2) below, highlights ten action points identified through cross-case analysis within the research team in discussion with PPI co-researchers, regional and national stakeholders and our advisory group and conceptually underpinned by NPT. As IMPRESS is an AR project, possible actions and solutions to problems of embedding PPI may be further considered heuristically; ‘tried out’ and ‘fine-tuned’ through various iterations in further cycles of AR in working towards sustainable PPI processes and outcomes for all stakeholders.

**Table 2:** PPI action framework for further embedding PPI within research programme.

NPT construct	Suggested action
**Making sense of PPI**	More training, education and advice and discussion around PPI
**Buying in to PPI**	More informal networking and cross-theme discussion on PPI sources and networks Support provided for PPI initiation before and maintenance between projects
**Doing PPI**	Plan PPI structures, purpose and roles in relation to project timelines Construct a CLAHRC EoE PPI resources handbook Greater transparency and sharing of project reports with PPI contributors CLAHRC to be flexibly responsive to changes in PPI plan within a project’s timescale Mobilise PPI for translating research into practice
**Appraising PPI**	Timely and appropriate feedback given to PPI contributors Emphasise the evaluation of PPI within projects beyond use of existing KPI tool.

## Discussion

The objectives of this first action research cycle of IMPRESS were to explore the experience of PPI within CLAHRC EoE, to identify barriers and enablers to “fully embedded, active and comprehensive PPI” in this research programme and to determine what is entailed in “normalising” a programme of PPI across a CLAHRC. The overall aim being to build knowledge and evidence but also build a framework to identify actions to improve research practice concerning PPI. Staley [16](2015) suggests that the impact of PPI is both highly context-dependent and reliant on the quality of specific PPI processes implemented. Our findings show that despite programme wide policy support and dedicated resource for PPI within the CLAHRC EoE programme and evidence of good PPI practice, PPI is not yet ‘fully embedded, active and comprehensive’ within this CLAHRC. The main facilitators and barriers to *making sense* of PPI, *buying into* PPI, *doing* PPI and *appraising* PPI within programme projects being identified in the summary sections above.

National realist PPI evaluations [21, 23] found key enabling contexts that influenced mechanisms for PPI were: research funder, topic and study design, host organisation, the lead investigator’s beliefs about PPI; and the extent to which there is a culture of involvement in the unit where the research takes place. Both studies also noted the importance of having one person per project team as the recognised leader or coordinator for PPI. IMPRESS findings detail how within this regional multi-disciplinary, multi-institutional programme where the funder is the same, the variable starting points in researchers’ knowledge of and experience with PPI and the existing regional PPI organisations and networks impact PPI. The lack of timely opportunities to share and further this learning across the programme, being a main barrier to its normalisation. Instead, sharing PPI experiences and guidance largely remained within-workplaces through formal presentations and informal discussion. As Hewlett et al., note [45](2006), when PPI contributors are often only present at formal meetings, such informal workplace discussions like ‘corridor meetings’, can impede full collaboration. The often short-term, short run-in nature of CLAHRC projects, coupled with the lack of identifying with any CLAHRC programme community, constrained possibilities of fostering a wider PPI learning culture. The constraints of this context have implications for NIHR national PPI policy [46] where CLAHRCs are anticipated to take a regional lead in developing and embedding PPI in research.

PPI collaborators interviewed for IMPRESS also did not stress having on person for leading PPI, as being important. This was perhaps because case study project teams were, in the main, comparatively small projects with few team members (not multi-site RCTs) and most PPI models employed were one-off consultations. Reflecting wider reported criticism and trends in PPI in healthcare improvement [43, 47], PPI in CLAHRC EoE’s project PPI models are largely concentrated at this ‘lower’ end of involvement continuum, where PPI is more in consultation than research leadership. IMPRESS findings did highlight the benefits of negotiating flexible PPI roles, (see also [10]) tailored to project and personal needs and for funding programmes to enable time and resources for this. Reflecting published commentaries on evaluating PPI [16], researchers interviewed for this study also often viewed PPI as too complex to evaluate objectively.

PPI in CLAHRC EoE has been a less distinct and transformative experience than that reported by in CLAHRC NWL where PPI was part of a novel ‘quality improvement sphere’ process supported with ongoing training across the programme. The experiences reported in EoE are more variable and corroborate more those of the CLAHRC NDL PPI evaluation [30], particularly so in regard to barriers to making sense of CLAHRCs themselves and their distinct PPI commitments:

‘…a sense of CLAHRC-related opacity pervades the interview transcripts […] CLAHRC is narrated as a somewhat occluded and impervious entity’ [30](p2701).

The reported mismatch between PPI contributors’ expected and actual levels of communication with project teams was also found in both this study and by Jordan et al. [30]p.2700. Although in CLAHRC NWL, it was health professionals in who ‘demanded evidence of PPI effects of the type typical in clinical practice, such as cost-effectiveness data, treating PPI as a discrete intervention to improve a specific health outcome’ [28](p69), in this CLAHRC it was non-clinical researchers who questioned the legitimacy of the PPI voice and PPI evidence base and hence requirements for PPI in their projects. The evaluation of the INVOLVE PPI standard [48] test-bed sites may shed further light on PPI processes embedded within NIHR research programmes and the projects and researchers that reside within them who operationalise PPI.

## Conclusions

Informed by NPT, this initial phase of the IMPRESS action research study explored in-depth, the experiences of researchers, PPI contributors and PPI stakeholders in the context of their mutual involvement in a regional programme of applied research. This approach has helped identify various barriers and facilitators to embedding PPI in a research programme and areas for future action to develop this further.

Trust, rapport and mutual respect builds in the early stages of action research and this supports the ongoing cycles of work [49]. We have so far drawn upon NPT retrospectively, post-implementation, but NPT can also be used to prospectively to support implementation of the further actions. Indeed some of the action points are already being addressed within the CLAHRC EoE in its funding the co-production of a PPI feedback tool [50], the co-production of a CLAHRC EoE PPI resources handbook [51] and in supporting training events.

In terms of limitations, the IMPRESS findings here cannot claim to be comprehensive being based on 10 out of 27 projects being live at the time of data collection. Being a ‘case study’ for IMPRESS may have led teams to reflect further upon and adapt their PPI during the data collection period, positively skewing results. The data collection window ensures that findings presented here are a snapshot in time rather than final outcomes of projects and their PPI. Findings and conclusions drawn here are based only on reported rather than directly observed processes such as observational data collection from research team meetings, and not all research team members were available or willing to be interviewed. Indeed, CLAHRC activities and partner organisations are often disparate and evolving, and balancing evaluation work with other responsibilities within the CLAHRC risks overburdening CLAHRC staff [52].

A strength of our findings is their triangulated and contextualised identification, of specific experiences of diverse project teams within the particular organisational context of applied health research in England. We have here identified and targeted actions to enable research programmes projects and participants to identify elements found in this context which facilitate and hinder PPI. These elements are likely to be recognised as applicable in other research programmes and as the results sections have highlighted, corroborate previous findings from CLAHRC NDL [30]. Case study project topics and aims were distinct and included diverse PPI contributors, and provide a basis from multiple perspectives for lessons learned to be shared with other research programmes and institutions wishing to embed PPI in their work.

## Supporting information

S1 Appendix NPT-informed topic guides for researchers and for PPI contributor interviews. (RTF)

S2 Appendix Final coding frame. (RTF)
