# Supplementary material for: ‘We’re passengers sailing in the same ship, but we have our own berths to sleep in’: Evaluating patient and public involvement within a regional research programme: An action research project informed by Normalisation Process Theory
Source: PLoS One. 2019 May 14;14(5):e0215953. doi: 10.1371/journal.pone.0215953 (PMC6516650; doi:10.1371/journal.pone.0215953)
Supplement: S1 Appendix — (RTF) [file pone.0215953.s001.rtf]

S1: Appendix NPT-informed topic guides for researchers and for PPI contributor interviews
IMPRESS INTERVIEW: C.I.s & RESEARCHERS 
(Left hand Column Numbers refer to Normalisation Process Theory (NPT) domain statements informing questions)
1.1


	Can you begin by telling me what you understand by the term 'Patient and Public Involvement (PPI) in research'?(Probe: other terms used, differentiation from participation as research subjects, differentiation from engagement/impact)

Can you tell me a little bit about whether patient and public involvement in research has been a feature of (your research/ research you have been involved in) prior to joining CLAHRC East of England? (Probe: Over how many years? What did this involve?)

Does patient and public involvement within the CLAHRC East of England differ in any way from your experience of PPI on other studies/research programmes and if so, how?	
1.2

	What is your understanding of the aims and objectives (or purpose and role) of PPI in research like yours? Across a research programme like the CLAHRC East of England?	
1.4


2.3	We are 'signed up' to deliver PPI but, in your opinion, how important is PPI in your research project/CLAHRC East of England?  (Probe: moral, methodological, policy value.  Could you imagine your study without PPI?)

Do you find your colleagues (on this study/other studies within the CLAHRC) share your view? (Probe: reasons for differences)	
2.1, 3.3


2.2, 3.1, 1.3

2.1, 3.3, 3.4	Do members of your research team have specific roles and responsibilities in relation to PPI? If so, what are they? (Probe: Is there someone in particular who is responsible for PPI in your research team, a PPI designer, named lead, liaison with theme PPI link?)

What do you think are the best ways to enable PPI to work well? What needs to be in place on a project? In a programme? (Probe: relationships, time, training, accessibility, budget etc.)

What support/ resource, if any, are provided to PPI members on your project (e.g. travel expenses, buddying, training, accessible times/ locations)? Is your budgeting for PPI sufficient/challenged?	
2.4, 3.1, 3.2	Have there been, or do you envisage there being any potential challenges in having PPI on your project? (Probe: time it takes, relationships, different PPI roles/strengths, maintaining PPI over course of study)
	
4.1


4.2 4.3


4.4	How do you judge whether PPI is making a difference to your project?
(Probe: formal or informal assessment of the PPI contribution to the study, making a difference as validating and/or changing research, examples of this, Key Performance Indicator (KPI) tool use here)

Do you share your evaluations of PPI working (or not working) with colleagues (on this study/other studies within the CLAHRC)? (Probe: time PPI takes, impact on other work activities)

Have you made any changes to the way that you deliver PPI within your project as it has developed? (Probe: reasons for changes, whether these have been documented)	

INDICATIVE TOPIC GUIDE IMPRESS INTERVIEW: PPI contributors

1.3


1.1


1.2


1.3
	Can you begin by telling me what your PPI role is on this XX project? (Probe: What PPI activities have you done/ will you be doing? Does/will this study take up much of your time? How much?) Why/How did you get involved in this project? Are there other PPI representatives on your project? (Probe: same/different roles, how well do they know one another)

What you understand by the term 'Patient and Public Involvement (PPI) in research'?(Probe: other terms used, differentiation from participation as research subjects, differentiation from engagement/impact)

Can you tell me a little bit about how long have you been involved in PPI in research?
(Probe: No. of years, no. and variety of projects/ PPI roles) 

Why do you think researchers ask service users, carers or members of the public to get involved as research partners? (What is the purpose and role of PPI?)

Your research project is one of a number in a funded programme of research called a CLAHRC, do you know much about CLAHRCs? Is PPI in a CLAHRC different? If so, how?	
1.4


2.3
	As a CLAHRC we are 'signed up' to deliver PPI but, in your opinion, how important is PPI in your research project/CLAHRC EoE?  (Probe: moral, methodological, policy value.  Could you imagine your study without PPI?)

Do you feel that researchers in your study really believe in PPI or see it as a 'tick box exercise' - something they 'have to do'? Why do you think this is/is not the case? Do you think other PPI members see themselves and their activities as being important to the research?	
2.1, 3.3


2.2, 3.1, 
3.3, 3.4


2.4	Who do you deal with most in the research team? (Probe: their job title, contact means, contact frequency) What about other members of the research team? 

Do you feel that you are given the support/ resources you need to carry out your role? (e.g. travel expenses, payment, childcare, emotional support, dietary requirements, accessible times/ locations/ training) Are these sufficient?

What is it that keeps you involved with this project?	
3.1, 3.2, 4.3	Have there been, or do you envisage their being any challenges for you in being involved with this project? 	
4.2 & 4.3


4.1


4.4
	Do you receive any feedback from researchers as to how your contributions, or those of fellow PPI members, have helped to develop the project? Can you give me any examples of the ways in which PPI has improved the study?

Has being involved with this research study been of benefit to you in any way? (Enjoyment, confidence etc.)

How does your research team judge whether PPI is making a difference to your project? (Probe: formal or informal assessment of the PPI contribution to the study)

Have you made any changes to the way that you do PPI within your project as it has developed? (Probe: reasons for changes, whether these have been documented)	
