# Supplementary material for: ‘We’re passengers sailing in the same ship, but we have our own berths to sleep in’: Evaluating patient and public involvement within a regional research programme: An action research project informed by Normalisation Process Theory
Source: PLoS One. 2019 May 14;14(5):e0215953. doi: 10.1371/journal.pone.0215953 (PMC6516650; doi:10.1371/journal.pone.0215953)
Supplement: S2 Appendix — (RTF) [file pone.0215953.s002.rtf]

S2: Appendix. IMPRESS Final coding frame 

Inductive codes:
A)	Description of the model of PPI on the project (may also be in NPT: coherence)
B)	Personal biography – this node includes accounts given by participants of their journey into PPI prior to their involvement or work on current study. 
C)	Reflections on the interview process – this node includes any instances where participants and/or interviewer openly reflect (in the transcript) on the interview/focus group process.
D)	Contextual sensitivities/tensions – this node includes any instance where the participant/interviewer are having to be politic in what they are saying around the research context (CLAHRC). N.B: This will usually warrant an accompanying annotation in Nvivo by the IMPRESS researcher.


There are a number of organisational levels, different projects and institutions being discussed in the transcripts, we need to make sure we are anonymising transcripts in systematic ways that make it clear which organisations we are referring to. We also need to annotate (highlight, right click, annotate) to differentiate where participants are talking about their CLAHRC case study project, and other projects that have included PPI.
Within NPT we are also coding both positive and negative components i.e., when things are/are not coherent, where there is /is not cognitive participation, where collective action is/is not happening, and finally where reflexive monitoring is/isn't taking place.


NPT constructs	NPT components	IMPRESS interpretation	Notes	
Coherence: This is the sense-making work/understanding that people do individually and collectively when they are faced with the problem of operationalizing PPI.	1.1 Differentiation: how a set of practices and their objects are different from each other. 


1.2 Communal specification: shared understanding of the aims, objectives, and expected benefits of a set of practices 

1.3 Individual specification: participants work in coherence as they need to do things that will help them understand their specific tasks and responsibilities around a set of practices. 
1.4 Internalization: understanding the value, benefits and importance of a set of practices.
	1.1	Differentiation: How the practices and procedures of PPI in the research field/CLAHRC etc. are/aren't different from participants' previous ways of doing PPI.

1.2	Communal specification: Shared/not shared understanding of the purpose of PPI across the CLAHRC and within projects.

1.3	Individual specification: Researchers and PPI members do/don't have a strong understanding of the PPI design and/or their role in PPI on project/PPI in the CLAHRC and what is expected of them.

1.4	Internalization: The potential or general worth participants do/don't attribute to PPI. 	Also here whether participants do/don't distinguish PPI from engagement, study participation, qualitative research etc.


N.B hard to spot in individual transcripts unless it is a 'we' script, or reflections on who on project does/doesn't understand purpose of PPI. 


1.4 subcodes: 
A) value of PPI for research 
B) value of PPI for themselves (prof. status, personal growth etc.) 
C) value of PPI for society/democracy etc.	
Cognitive Participation: This is the relational 'buy in' work that people do to build and sustain a community of practice around PPI.
	2.1 Initiation: whether or not key participants are actively working to drive new practices forward. 

2.2 Enrolment: participants organize or reorganize themselves and others so as to collectively contribute to the work involved in new practices. This is complex work that may involve rethinking individual and group relationships between people and things. 
2.3 Legitimation: ensuring that participants believe it is right for them to be involved in an innovation, and that they can make a valid contribution to it. 
2.4 Activation: Once it is underway, participants need to collectively define the actions and procedures needed to sustain a practice and to stay involved. 
	2.1 Initiation: Key individuals do/don't drive PPI forward, could be the PI, an assigned coordinator, PPI organisation etc.

2.2 Enrolment: Are/aren't research teams/themes set up adequately in order to deliver their PPI? 


2.3 Legitimation: Are/aren't research teams/themes committed to PPI and understand how they can contribute to it? A) Keen to do the research B) Keen to do PPI

2.4 Activation: Are/aren't members keeping PPI in view and connecting actions with the people who need to be doing them to sustain PPI.	E.g. setting up PPI systems, procedures, and protocols and engaging with others to make PPI happen is often delegated to researchers. Are the lead investigators supporting this? 

Have/haven't they made the necessary adjustments? Do/don't participants agree that PPI should be part of their work?


E.g. statements where interviewees express a conviction or lack of conviction towards PPI, or justify their expertise in it.


E.g. instances where researchers are/aren't keeping PPI on track, where PPI members might be prompting the team.	
Collective Action: This is the operational 'doing' work that people do to enact PPI.
	3.1 Interactional Workability: the interactional work that people do when they seek to operationalize innovations in everyday settings. 
3.2 Relational Integration: the knowledge work that people do to build accountability and maintain confidence in a set of practices and in each other, as they use those practices. 
3.3 Skill Set Workability: the allocation work that underpins the division of labour that is built up around a set of practices as they are being operationalized within the real world. 
3.4 Contextual Integration: the resource work - managing a set of practices through allocating different kinds of resources and the execution of protocols, policies and procedures. 
	3.1 Interactional Workability: The work that people do/don't do when they seek to make PPI happen in the project/HEI etc.


3.2 Relational Integration: The work participants do/don't do to maintain confidence and trust in one another and their PPI processes/roles.


3.3 Skill Set Workability: The PPI work/tasks are/aren't appropriately allocated and/or people are/aren't supported to do it.


3.4 Contextual Integration: PPI is/isn't adequately resourced by institutions/funders/the project 
	E.g. sorting out payments for PPI, doing the lay summary review, giving feedback, answering e-mails, setting up/attending meetings (or not).

E.g. do/don't express confidence/trust in one another 'he/she is always makes useful points' 'the CI gave me quite cursory feedback' ' I felt I could speak openly' 


E.g. has the right training been given, are people given enough time to do tasks, are meetings which involve PPI members held at accessible times/locations.


E.g. Are university/trust/CLAHRC systems for managing PPI recruitment/payments appropriate? Are CLAHRC projects allocating 5% of their budgets to PPI? How is this policy working out?	
Reflexive Monitoring: the appraisal work that people do to assess and understand the ways that working with PPI affects them and others around them.	4.1 Systematization: participants in any set of practices may seek to determine how effective and useful it is for them and for others, and this involves the work of collecting information in a variety of ways. 
4.2 Communal appraisal: participants work together to evaluate the worth of a set of practices. 
4.3 Individual appraisal: participants in a new set of practices also work within their experience as individuals to appraise its effects on themselves and those contexts in which they are set. Their appraisal work leads to actions through which individuals express their personal relationships to new technologies or complex interventions. 
4.4 Reconfiguration: appraisal work by individuals or groups may lead to attempts to redefine procedures or modify practices - and even to change the shape of a new technology itself.  	4.1 Systematization: Do/don't research teams/themes seek to determine how effective and useful their PPI is? 


4.2 Communal appraisal: Collective +/- evaluations, do/don't research teams/themes ask each other 'is PPI working?' 


4.3 Individual appraisal: Individual actual (not potential 1.4) +/- evaluations of PPI for themselves, the project, or the CLAHRC


4.4 Reconfiguration: Do/don't research teams/themes' appraisals lead to any actual changes to the way they do PPI/research. 	E.g. Do/don't they have any formal/informal mechanisms, policies, processes, tracking or evaluation for assessing PPI effectiveness?


'we' script, 'we all thought that bit went really well', 'we agreed that was too much for him/her' 


4.3 subcodes
A) +/- for research
B) +/- for PPI processes
C) +/- for themselves, personally
D) +/- for society


4.3 Subcodes 
A) potential/suggested changes to PPI or research that may happen, but haven't yet. B) actual changes/'tweaks' to PPI or research (impact) within project's lifetime.	
